# lncRNA OXCT1-AS1 Promotes Metastasis in Non-Small-Cell Lung Cancer by Stabilizing LEF1, *In Vitro* and *In Vivo*

**DOI:** 10.1155/2021/4959381

**Published:** 2021-07-21

**Authors:** Binru Li, Libo Zhu, Linlin Li, Rui Ma

**Affiliations:** Department of Thoracic Medicine, Cancer Hospital of China Medical University, Liaoning Cancer Hospital and Institute, Shenyang, Liaoning 110042, China

## Abstract

Long noncoding RNAs (lncRNAs) play nonnegligible roles in the metastasis of non-small-cell lung cancer (NSCLC). This study is aimed at investigating the biological role of lncRNA OXCT1-AS1 in NSCLC metastasis and the underlying regulatory mechanisms. The expression profiles of lncRNA OXCT1-AS1 in different NSCLC cell lines were examined. Then, the biological function of lncRNA OXCT1-AS1 in NSCLC metastasis was explored by loss-of-function assays *in vitro* and *in vivo*. Further, the protective effect of lncRNA OXCT1-AS1 on lymphoid enhancer factor 1 (LEF1) was examined using RNA pull-down and RNA immunoprecipitation assays. Additionally, the role of LEF1 in NSCLC metastasis was investigated. Results indicated that lncRNA OXCT1-AS1 expression was significantly increased in NSCLC cell lines. Functional analysis revealed that knockdown of lncRNA OXCT1-AS1 impaired invasion and migration *in vitro*. Additionally, the ability of lncRNA OXCT1-AS1 to promote NSCLC metastasis was also confirmed *in vivo*. Mechanistically, through direct interaction, lncRNA OXCT1-AS1 maintained LEF1 stability by blocking NARF-mediated ubiquitination. Furthermore, LEF1 knockdown impaired invasion and migration of NSCLC *in vitro* and *in vivo*. Collectively, these data highlight the ability of lncRNA OXCT1-AS1 to promote NSCLC metastasis by stabilizing LEF1 and suggest that lncRNA OXCT1-AS1 represents a novel therapeutic target in NSCLC.

## 1. Introduction

Non-small-cell lung cancer (NSCLC) is the main type of lung cancer and one of the most frequently occurring cancers worldwide [1]. According to the Global Cancer Statistics 2018, lung cancer cases account for about 11.6% of the total number of cancer diagnoses; furthermore, among the total number of cancer-related deaths, 18.4% are attributable to lung cancer [2]. Although progress has been made in NSCLC treatment, metastatic NSCLC is still associated with a high rate of mortality [3, 4], indicating the critical need for the identification of new therapeutic targets. Metastasis is a complex multistep process, and the invasion and migration capability of cancer cells determines the metastasis of NSCLC [5, 6]. Furthermore, a gain of mesenchymal characteristics as well as loss of epithelial features frequently accompany the attainment of metastatic ability in NSCLC; this epithelial-mesenchymal transition involves numerous molecules such as long noncoding RNA (lncRNA), transcription factor, and circular RNA [7, 8]. However, the precise mechanisms underlying NSCLC metastasis have not yet been identified.

lncRNA is a class of RNA without protein-coding functions. Emerging researches have shown that many lncRNAs are dysregulated in NSCLC, and that their dysregulation contributes to metastasis in cancer [9, 10]. Recently, Liao et al. [11] found that the lncRNA CCHE1 increased the proliferation, metastasis, and invasion of NSCLC cells, and was predictive of poor survival in NSCLC patients. Chen et al. [12] identified a novel lncRNA OXCT1-AS1 through microarray experiments, and showed that its expression was elevated in lymph node metastasis. Furthermore, these authors found that lncRNA OXCT1-AS1 suppresses miR-455-5p to promote bladder cancer proliferation and invasion. However, the exact role of lncRNA OXCT1-AS1 in the metastasis of NSCLC, and the mechanisms involved, remain poorly understood.

Growing evidence indicates that lncRNAs participate in the regulation of gene expression at the transcription or posttranscription levels, as well as that of pathological and cellular physiological processes [13, 14]. In addition, lncRNAs regulate protein stability [15], as competing endogenous RNAs [16], and also regulate mRNAs' stability [17]. Jiang et al. [18] recently reported that lncRNA SNHG15 interacts with and stabilizes Slug by blocking its ubiquitination to promote colon cancer progression. Furthermore, Xue et al. [15] found that lncRNA LINRIS blocked ubiquitination of IGF2BP2 in colorectal cancer, maintaining its stability. Therefore, we speculated that lncRNA OXCT1-AS1 may play a potential role in migration, invasion, and metastasis of NSCLC by influencing the stability of target protein.

Lymphoid enhancer-binding factor 1 (LEF1), which is characterized by a high expression in tumors, is a key transcription factor of the WNT pathway and participates in the regulation of cell metastasis and proliferation [19]. Nguyen et al. [20] found that the WNT/TCF pathway through HOXB9 and LEF1 mediates metastasis in lung adenocarcinoma. Furthermore, results from Zhao et al.'s study [21] revealed that in esophageal squamous cell carcinoma, elevated level of LEF1 was obviously associated with lymph node metastasis, histologic grade, TNM stage, and poor prognosis. Therefore, we speculate that lncRNA OXCT1-AS1 may promote NSCLC metastasis by stabilizing LEF1.

To the best of our knowledge, the present study is the first to profile the expression of lncRNA OXCT1-AS1 in NSCLC cells. Additionally, we explored the role of lncRNA OXCT1-AS1 in regulating migration, invasion, and metastasis of NSCLC and investigated the interactions between lncRNA OXCT1-AS1 and LEF1, with the aim of identifying novel therapeutic targets for metastatic NSCLC.

## 2. Methods

### 2.1. Cell Culture

Human normal epithelial cell line BEAS-2B and human NSCLC cell lines (A549, H1299, H23, and HCC827) were obtained from the American Type Culture Collection (Manassas, VA, USA). NSCLC cell lines were cultured in RPMI 1640 medium (Thermo Fisher Scientific, Inc., Waltham, MA, USA) adding 10% fetal bovine serum (FBS; Thermo Fisher Scientific). BEAS-2B cells were cultured in BEGM medium (Thermo Fisher Scientific) adding 10% FBS. All cells were incubated in a humidified incubator at 37°C with 5% CO_2_ and saturated humidity. When cell confluence had reached 70%–80%, the cells were detached with trypsin (0.25%) to obtain a single-cell suspension.

### 2.2. Lentiviral Vector System, Plasmids, and Cell Transfection

shRNAs targeting lncRNA OXCT1-AS1 (defined as sh-lncRNA) or LEF1 mRNA (defined as sh-LEF1) were ligated into the vector. The empty vector, which was used as the negative control, was named sh-NC. The lentiviruses were synthesized by Wuhan GeneCreate Biological Engineering Co., Ltd. (Wuhan, Hubei, China). Cells were transfected with these lentiviruses. To obtain stably transfected cell lines, H1299 and A549 cells were treated with 2–3 *μ*g/mL puromycin (14 days). Knockdown efficiency was evaluated by qRT-PCR and western blot analyses. The expression vectors for Flag-tagged MS2 coat protein (MCP) and MS2-tagged lncRNA OXCT1-AS1, NARF, and LEF1 were provided by GeneCreate Biological Engineering, and Flag-tagged expression vectors for full-length lncRNA OXCT1-AS1, full-length LEF1 mRNA, and lncRNA OXCT1-AS1 fragments were provided by Sangon Biotech Co., Ltd. (Shanghai, China). The plasmids were transfected into A549 cells with Lipofectamine 3000 (Thermo Fisher Scientific), as recommended by the manufacturer. Transfection efficiency was assessed by qRT-PCR and western blot analyses. The shRNAs' sequences are as follows: lncRNA OXCT1-AS1—forward, 5′-CAC CGC TTA CAT AGA GTA AGT TTG CCG AAG CAA ACT TAC TCT ATG TAA GC-3′; reverse, 5′-AAA AGC TTA CAT AGA GTA AGT TTG CTT CGG CAA ACT TAC TCT ATG TAA GC-3′; LEF1—forward, 5′-CAC CGC CTT AAA TCT ACG CAG AAG ACG AAT CTT CTG CGT AGA TTT AAG GC-3′; reverse, 5′-AAA AGC CTT AAA TCT ACG CAG AAG ATT CGT CTT CTG CGT AGA TTT AAG GC-3′; NARF—forward, 5′-CAC CGC GTT CTG AAC CTT AAC AAG ACG AAT CTT GTT AAG GTT CAG AAC GC-3′; reverse, 5′-AAA AGC GTT CTG AAC CTT AAC AAG ATT CGT CTT GTT AAG GTT CAG AAC GC-3′.

### 2.3. RNA Extraction and qRT-PCR Analysis

Total RNA was extracted from cells of both cell lines using a Total RNA extraction kit (Beyotime Institute of Biotechnology, Shanghai, China). The primer sequences for lncRNA OXCT1-AS1, GAPDH, and LEF1 were designed and synthesized by Sangon Biotech Co., Ltd. Total RNA was reverse transcribed into cDNA using a Reverse Transcriptase Kit (Beyotime Institute of Biotechnology), as recommended by the manufacturer. Then, qRT-PCR was performed using SYBR Premix Ex Taq II (Takara Biotechnology Co., Ltd., Dalian, Liaoning, China). The primer sequences were as follows: lncRNA OXCT1-AS1—forward, 5′-CTG GAC TGC GTT CAC GTT TC-3′; reverse, 5′-CTG GAC TGC GTT CAC GTT TC-3′; LEF1—forward, 5′-AGC GAA TGT CGT TGC TGA GTG-3′; reverse, 5′-CTC TTG CAG ACC AGC CTG GAT AA-3′; GAPDH—forward, 5′-GCA TCC TGG GCT ACA CTG-3′; reverse, 5′-TGG TCG TTG AGG GCA AT-3′. PCR was performed in a reaction mix (10 *μ*L), and the protocol consisted of a denaturation step at 95°C (30 s), followed by amplification with 40 cycles at 95°C (5 s) and 60°C (30 s); this was followed by a melt curve step at 65°C to 95°C, with temperature increments of 0.5°C for 5 s. GAPDH was used as the endogenous control. Gene expression data were analyzed with the 2^−ΔΔCt^ method [22].

### 2.4. Isolation of Nuclear and Cytoplasmic Fractions

In accordance with the manufacturer's protocol of the Nuclear and Cytoplasmic Extraction Reagent Kit (Beyotime Institute of Biotechnology), nuclear-cytoplasmic fractionation was conducted. Briefly, after washing in PBS, cells were suspended in cytoplasmic extraction reagent I (0.2 mL), followed by the addition of cytoplasmic extraction reagent II (11 *μ*L). The suspension was then incubated on ice for 60 s, followed by centrifugation (16,000 g, 5 min). The cytoplasmic extract was the supernatant fraction, and the crude nuclei formed the pellet fraction.

### 2.5. Transwell Assays and Cell Proliferation Assays

For migration, 10,000 cells were placed into the upper chamber (8 *μ*m pore size) (Corning Incorporated, Corning, New York, USA). For invasion, 1 × 10^5^ cells were placed into the upper chamber with Matrigel (Corning Incorporated). RPMI 1640 medium (10% FBS) was supplemented to the lower chamber. After incubation (24 h), the cells were removed. Then, cells through the membrane were fixed with methanol, followed by crystal violet staining (0.1%). Images were acquired, and then cell numbers were counted under an inverted microscope (Olympus Corporation, Tokyo, Japan). For assessment of cell proliferation, 3000 cells were plated in 96-well plate. After incubation for different times (24, 48, and 72 h), cell viability was determined by the addition of CCK-8 (10 *μ*L), using a CCK-8 kit (Dojindo, Mashikimachi, Kumamoto, Japan). The absorbance (450 nm) was measured using a microplate reader (Beijing Potenov Technology Co., Ltd., Beijing, China).

### 2.6. Western Blot Analysis

Using a RIPA kit (Beyotime), total protein was extracted. After protein lysis and centrifugation, the protein concentration was detected by a bicinchoninic acid kit (Beyotime Institute of Biotechnology). Subsequently, cell lysates were separated by SDS-PAGE and transferred onto PVDF membranes. After blocking with 5% skim milk (60 min), it was probed with primary antibodies overnight (4°C). After washing with PBST (4 times), secondary antibodies were added for 60 min (25°C). After washing again with PBST (4 times), enhanced chemiluminescence substrates (Beyotime Institute of Biotechnology) were applied to visualize the protein bands in a dark room. Quantification of protein bands was performed by ImageJ software (National Institutes of Health, Bethesda, MD, USA). The relative protein expression was normalized to *β*-actin. The following antibodies were used: Mouse Anti-E-cadherin antibody (ab231303, Abcam, Cambridge Biomedical Campus, Cambridge, UK; 1/1000), Mouse Anti-N-cadherin antibody (ab98952, Abcam, 1/1000), Rabbit Anti-vimentin antibody (ab137321, Abcam, 1/2000), Rabbit Anti-Snail antibody (ab216347, Abcam, 1/1000), Rabbit Anti-LEF1 antibody (ab137872, Abcam, 1/2000), Mouse Anti-SIRT1 antibody (ab110304, Abcam), Mouse Anti-Flag tag antibody (ab125243, Abcam), Rabbit Anti-NARFL antibody (ab241215, Abcam, 1/1500), Mouse Anti-HA tag antibody (ab18181, Abcam, 1/1500), and Rabbit Anti-*β*-actin antibody (ab179467, Abcam, 1/5000), Goat Anti-Mouse IgG H&L (HRP) (ab205719, Abcam, 1/50,000), and Goat Anti-Rabbit IgG H&L (HRP) (ab205718, Abcam, 1/50,000).

### 2.7. Database Analysis

The targets of lncRNA OXCT1-AS1 were acquired through the RNA Interactome Database (http://www.rna-society.org/rnainter/home.html/) and PRIdictor websites (http://bclab.inha.ac.kr/pridictor/pridictor.html). Through the Protein Lysine Modifications Database (PLMD, http://plmd.biocuckoo.org/), lysine modification sites were acquired.

### 2.8. RNA Pull-Down Assays

Biotinylated lncRNA OXCT1-AS1, antisense lncRNA OXCT1-AS1, and lncRNA OXCT1-AS1 fragments were transcribed in A549 and 293T cells. The RNA products treated with RNase-free DNase I (Thermo Fisher Scientific) were purified using a Total RNA Purification Kit (Sangon Biotech Co., Ltd.). Then, 4 *μ*g of biotin-labelled RNAs were denatured (65°C, 300 s) in PA buffer and slowly cooled. Next, the folded RNA was supplemented with 2 U/mL RNasin (Solarbio) and streptavidin Dynabeads (Thermo Fisher Scientific) and then incubated for 60 min at 4°C. After washing with wash buffer (4 × 5 min) and preclearing using streptavidin Dynabeads, the protein lysate was incubated with the folded RNA-bead complex and 20 *μ*g/mL yeast tRNA at 4°C for 210 min. After washing, beads were boiled in 40 *μ*L 1x SDS loading buffer for 600 s. Then, lncRNA OXCT1-AS1 interacting proteins were subjected to western blot analysis.

### 2.9. RNA Immunoprecipitation Assay

A549 cells were lysed in 500 *μ*L of RNA immunoprecipitation buffer (Beyotime Institute of Biotechnology) containing protease inhibitors and an RNAase inhibitor. After centrifugation (8,000 g, 600 s). The supernatants were incubated with anti-Flag, anti-LEF1, anti-mouse IgG, or anti-rabbit IgG antibody for 120 min at 4°C (gentle rotation). Then, protein A/G beads (40 mL) were added and incubated for 60 min (4°C). After washing in RIPA buffer (3 times) and PBS (once), RNA was extracted and qRT-PCR was performed to detect the level of gene expression.

### 2.10. Coimmunoprecipitation (CO-IP) Assay

A549 cells were lysed in 500 *μ*L of RNA immunoprecipitation buffer (Beyotime Institute of Biotechnology) containing protease inhibitors. After preclearing with 30 *μ*L protein G/A-plus agarose beads (Thermo Fisher Scientific) for 60 min (4°C), centrifugation was performed (3,000 g, 5 min, 4°C). Supernatants were incubated with antibody (2 *μ*g) for 240 min (4°C). Then, the immunoprecipitates were incubated with protein G/A-plus agarose beads (30 *μ*L) overnight. After centrifugation (3,000 g, 5 min, 4°C), the precipitates were washed (5 × 10 min) with bead wash solution, followed by resuspension in loading buffer (60 *μ*L) and incubation for 300–600 s (100°C). Next, western blot was performed.

### 2.11. In Vivo Tumorigenesis in Nude Mice

Animal studies were implemented following the principles and procedures of the National Institutes of Health Guide for the Care and Use of Animals. Four-to-five-week-old BALB/c-nude mice (50% male and 50% female) were obtained from the Laboratory Animal Center of Jilin University (SCXK (Ji) 2016-0001) (Changchun, Jilin, China). The research procedures were reviewed and approved by the Medical Ethics Committee of China Medical University. Mice were allowed free access to food as well as water and maintained at 20 ± 2°C. For *in vivo* assay of cancer cell metastasis (ten mice per group), negative control or lncRNA OXCT1-AS1- (or LEF1-) knockdown A549 cell suspensions (1 × 10^6^ cells, 100 *μ*L) were injected into the tail veins of nude mice. BALB/c-nude mice were euthanized as soon as the following symptoms were detected: (a) inability to obtain water or food; (b) general lack of moving activities; (c) severe cachexia (weight loss approaching 25%); (d) pale appearance, body coat looking scruffy and unhealthy; (e) infection at the injection site; and (f) breathing problem. After 3 weeks, BALB/c-nude mice were anesthetized with 1% isoflurane and sacrificed by decapitation. Their lungs were harvested for hematoxylin and eosin (H&E) staining. The lung nodules were quantified microscopically by three observers. The body weights of nude mice were measured regularly.

### 2.12. Statistical Analysis

Each experiment was repeated in triplicate independent experiments. The data are shown as the mean ± SEM. GraphPad software 8 (GraphPad Software, Inc., La Jolla, CA, USA) and SPSS (IBM Corp., Armonk, NY, USA) were used for statistical analyses. Student's *t*-test was performed to evaluate the difference between two groups. The significance of the difference between multiple groups was analyzed by one-way analysis of variance followed by Tukey's post hoc test. Values of *P* < 0.05 were considered to indicate statistical significance. ^∗^*P* < 0.05 and ^∗∗^*P* < 0.01 compared with BEAS-2B or sh-NC cells. ^#^*P* < 0.05 and ^##^*P* < 0.01 compared with MG132 treatment.

## 3. Results

### 3.1. lncRNA OXCT1-AS1 Is Upregulated in NSCLC Cell Lines

**Figure 1:**
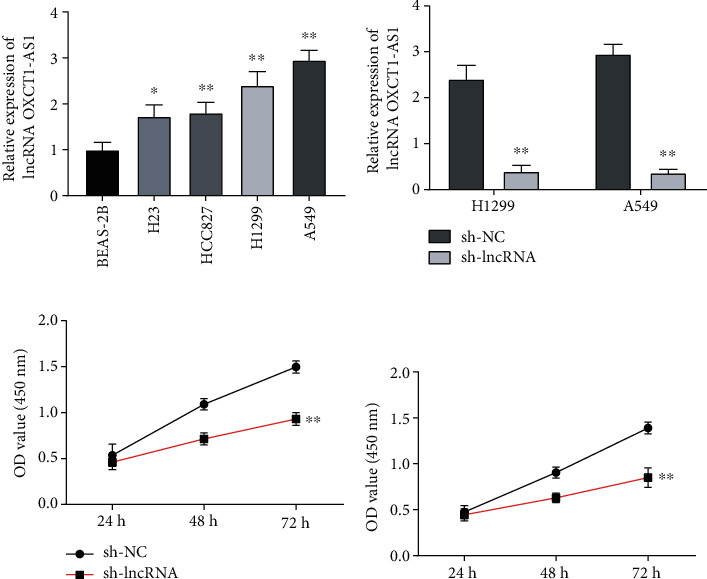
lncRNA OXCT1-AS1 is highly expressed in NSCLC. (a) The expression of lncRNA OXCT1-AS1 in a normal human lung epithelial cell line (BEAS-2B) and four NSCLC cell lines (H23, HCC827, H1299, and A549) was detected by qRT-PCR. (b) lncRNA OXCT1-AS1 expression in lncRNA stably depleted (sh-lncRNA) and negative control (sh-NC) H1299 and A549 cells was measured by qRT-PCR. Cell proliferation of sh-lncRNA and sh-NC H1299 cells (c) or A549 cells (d) was measured by CCK-8 assays.

In order to obtain lncRNA OXCT1-AS1 expression profiles in NSCLC, lncRNA OXCT1-AS1 levels were measured in different NSCLC cell lines via qRT-PCR assays. As shown in Figure 1(a), lncRNA OXCT1-AS1 levels were significantly elevated in NSCLC cells (H23, HCC827, H1299, and A549) compared with that in BEAS-2B cells; among these cancer cell lines, H1299 and A549 cells showed the highest upregulation of lncRNA OXCT1-AS1 expression. sh-lncRNA was synthesized to knock down the expression of lncRNA OXCT1-AS1, and successful interference was observed in H1299 and A549 cells relative to sh-NC (*P* < 0.01), as shown in Figure 1(b). Cell proliferation curves were drawn at different time points, and CCK-8 assays demonstrated that the viability of H1299 cells transfected with shRNA was obviously lower than that of the sh-NC group cells (*P* < 0.01; Figure 1(c)). Similar results were obtained in A549 cells (*P* < 0.01; Figure 1(d)).

### 3.2. Knockdown of lncRNA OXCT1-AS1 Suppressed Migration and Invasion of NSCLC Cells

**Figure 2:**
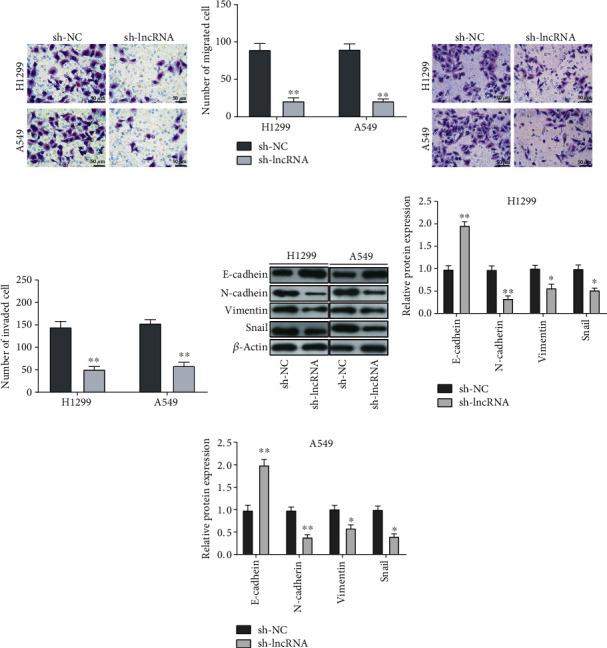
lncRNA OXCT1-AS1 knockdown decreased the migration and invasion of NSCLC cells. (a, b) Transwell assays were performed to assess the migration of H1299 and A549 cells. Quantitative data were from 5 fields; scale bar = 50 *μ*m. (c, d) Transwell assays were performed to assess the invasion of H1299 and A549 cells. Quantitative data were from 5 fields; scale bar = 50 *μ*m. (e–g) Western blot was performed to measure the level of E-cadherin, N-cadherin, vimentin, and Snail in H1299 and A549 cells, as normalized to the *β*-actin level.

To evaluate the effects of lncRNA OXCT1-AS1 on cell migration and invasion, *in vitro* function assays were conducted. Transwell assay results demonstrated that the migration ability of H1299 and A549 cells was markedly impaired by lncRNA knockdown (*P* < 0.01; Figures 2(a) and 2(b)). In addition, downregulation of lncRNA OXCT1-AS1 distinctly decreased the invasion ability of H1299 and A549 cells (*P* < 0.01; Figures 2(c) and 2(d)). Further, the expression of proteins associated with epithelial-mesenchymal transition was determined through western blot analysis. Results indicated higher expression of E-cadherin and weaker expression of N-cadherin, Snail, and Vimentin in the sh-lncRNA group compared with that in the sh-NC group (Figures 2(e)–2(g)).

### 3.3. Knockdown of lncRNA OXCT1-AS1 Suppressed NSCLC Metastasis

**Figure 3:**
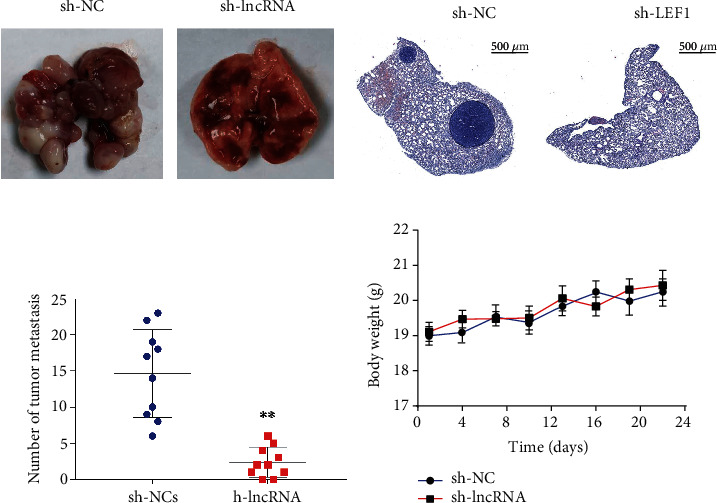
lncRNA OXCT1-AS1 knockdown decreased NSCLC metastasis. (a) The images of metastatic nodules in the lungs after tail vein injection with lncRNA OXCT1-AS1-downregulated and negative control A549 cells. (b) H&E staining section of the lungs in nude mice after tail vein injection with lncRNA OXCT1-AS1-downregulated and negative control A549 cells. (c) The average number of metastatic nodules in the lungs in the tail vein injection model. (d) The body weights of mice were measured.

To further confirm the effects of lncRNA OXCT1-AS1 on metastasis *in vivo*, lncRNA OXCT1-AS1-downregulated and negative control A549 cells were injected into the tail vein of nude mice. As shown in Figures 3(a)–3(c), lncRNA OXCT1-AS1 depletion dramatically suppressed the development of pulmonary metastasis. H&E staining indicated that the number of metastatic nodules in the lung was significantly lower in the sh-lncRNA group compared with that in the sh-NC group (Figures 3(b) and 3(c)). Interestingly, the body weights of mice were measured and were shown to be stable (Figure 3(d)). These results demonstrate that lncRNA OXCT1-AS1 might act as a key factor to promote metastasis in NSCLC.

### 3.4. lncRNA OXCT1-AS1 Interacts with LEF1 and Upregulates Its Expression

**Figure 4:**
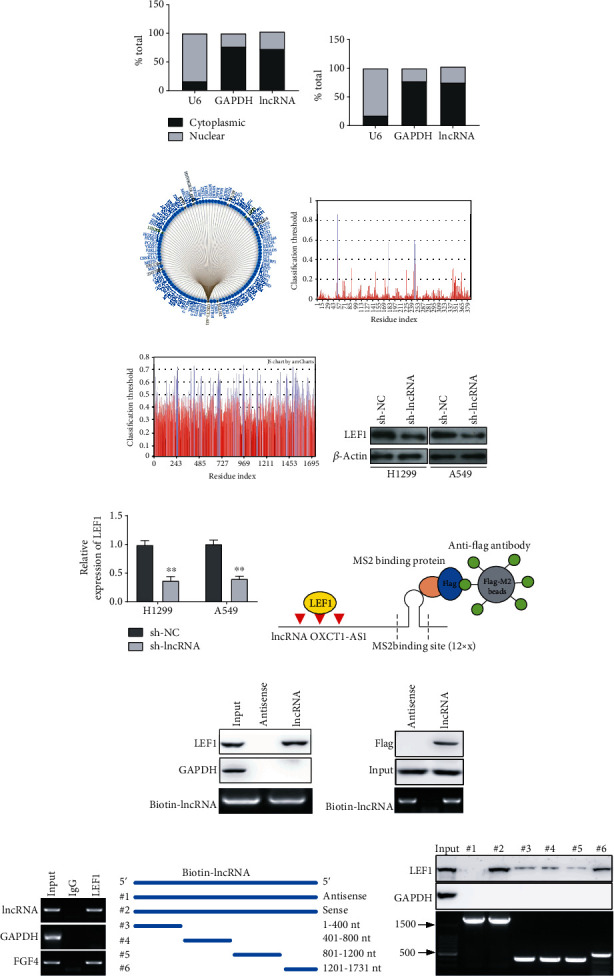
lncRNA OXCT1-AS1 interacts with LEF1 in NSCLC cells. Subcellular fractionation assay and qRT-PCR were carried out to evaluate the distribution of lncRNA OXCT1-AS1 in the nucleus and cytoplasm of the H1299 (a) and A549 cells (b). (c) The downstream targets were predicted using the RNAInter website. The binding sites of lncRNA OXCT1-AS1 in LEF1 (d) and the binding sites of LEF1 in lncRNA OXCT1-AS1 (e) were predicted using PRIdictor. (f, g) LEF1 protein expression in lncRNA OXCT1-AS1 stably depleted and negative control H1299 and A549 cells was measured by western blot. (h) Schematic presentation of the MS2-RNA pull-down strategy used to assess the interaction between LEF1 and lncRNA OXCT1-AS1. (i) Whole-cell lysates of A549 cells transfected with antisense-lncRNA-MS2 or lncRNA-MS2 were incubated with amylose beads and MBP-MS2 purified protein, followed by western blot analysis. GAPDH served as a negative control. (j) RNA pull-down assay using 293T cells cotransfected with Flag-LEF1 and lncRNA OXCT1-AS1-MS2 or antisense lncRNA OXCT1-AS1-MS2, followed by western blot analysis. (k) RNA immunoprecipitation assays were performed in A549 cell extracts using a LEF1 antibody, followed by qRT-PCR. GAPDH mRNA and FGF4 mRNA were used as the negative and positive control, respectively. (l) Deletion mapping of the LEF1-binding domain in lncRNA OCT1-AS1. Diagrams of full-length lncRNA OXCT1-AS1 and the deletion fragments. (m) Immunoblot analysis of LEF1 in A549 cells pulled down using different lncRNA OXCT1-AS1 fragments. GAPDH was used as the negative control. The bottom image shows each lncRNA OXCT1-AS1 fragment.

To identify the mechanism underlying lncRNA OXCT1-AS1-induced metastasis in NSCLC, the distribution of lncRNA OXCT1-AS1 was first investigated in H1299 and A549 cells. The results shown in Figures 4(a) and 4(b) indicated that lncRNA OXCT1-AS1 was enriched in the cytoplasm. Then, downstream targets of lncRNA OXCT1-AS1 were predicted using bioinformatics databases. As shown in Figure 4(c), among the 429 downstream targets, transcription factor LEF1, which was identified using the RNAInter (http://www.rna-society.org/rnainter/home.html/) and PRIdictor websites (http://bclab.http//inha.ac.kr/pridictor/pridictor.html/), was capable of binding lncRNA OXCT1-AS1. The binding sites on LEF1 and lncRNA OXCT1-AS1 are shown in Figures 4(d) and 4(e). Next, we explored the relationship between lncRNA OXCT1-AS1 and LEF1, and found that lncRNA OXCT1-AS1 knockdown attenuated LEF1 protein expression in H1299 and A549 cells (Figures 4(f) and 4(g)). To confirm the interaction between lncRNA OXCT1-AS1 and LEF1 protein, RNA pull-down assays were performed in A549 cells. The results manifested that lncRNA OXCT1-AS1 pulled down LEF1 but not GAPDH (Figures 4(h) and 4(i)). Additionally, RNA pull-down assays in 293T cells transfected with Flag-LEF1, lncRNA OXCT1-AS1, or its antisense lncRNA revealed an ability of lncRNA OXCT1-AS1 to pull down Flag-LEF1 (Figure 4(j)). To further validate this interaction between lncRNA OXCT1-AS1 and LEF1, RNA immunoprecipitation assays were performed. As Figure 4(k) displays, LEF1 was associated with lncRNA OXCT1-AS1 but not control GAPDH mRNA. Deletion-mapping analysis indicated that the lncRNA OXCT1-AS1 fragments 1201 nt and 1731 nt were involved in the interaction with LEF1 (Figures 4(l) and 4(m)). We conclusively demonstrate that lncRNA OXCT1-AS1 interacts with LEF1 in NSCLC cells.

### 3.5. lncRNA OXCT1-AS1 Stabilizes LEF1 by Blocking NARF-Mediated Ubiquitination

**Figure 5:**
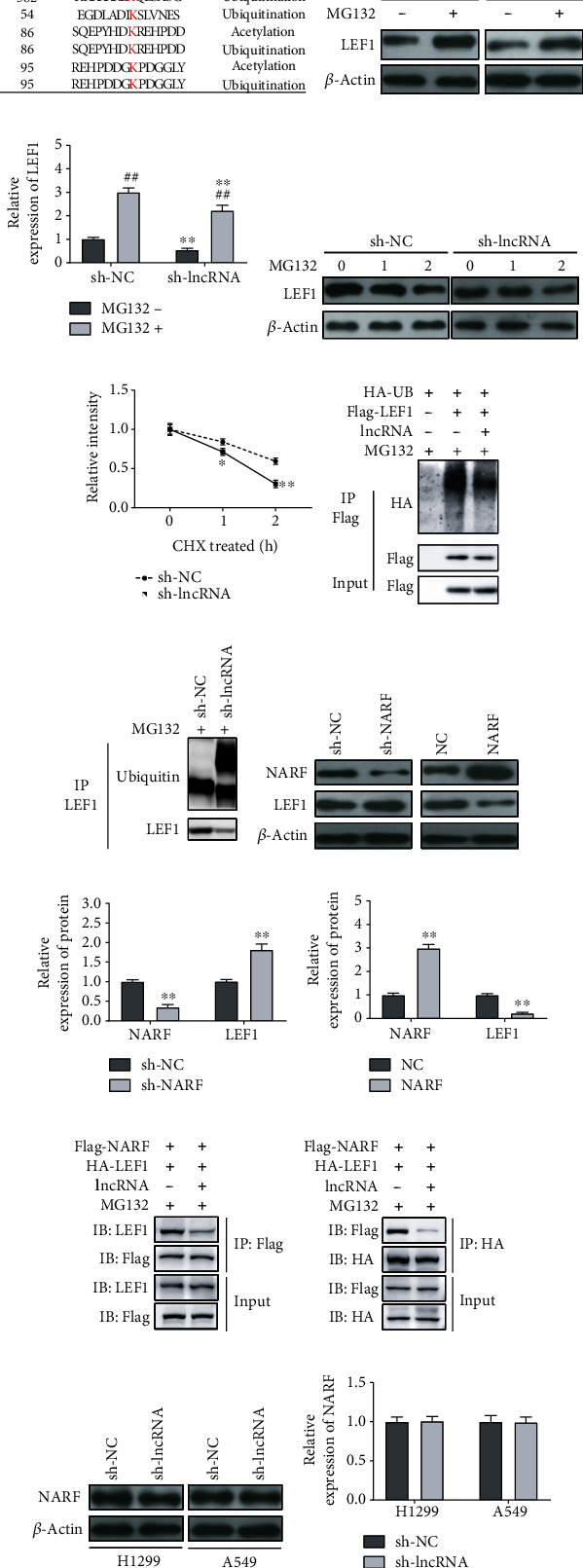
lncRNA OXCT1-AS1 stabilizes LEF1 by inhibiting NARF-mediated LEF1 ubiquitination. (a) The ubiquitination sites of LEF1 were predicted using Protein Lysine Modifications Database. (b, c) A549 cells were transfected with shRNA against lncRNA OCT1-AS1, followed by MG132 treatment. Then, LEF1 expression was detected by western blot analysis. (d, e) A549 cells were transfected with shRNA against lncRNA OCT1-AS1, followed by CHX treatment (100 *μ*g/mL) for different times (0, 1, and 2 h). Then, LEF1 expression was detected by western blot analysis. (f) After transfection with HA-UB, Flag-LEF1, or lncRNA OCT1-AS1 in combination or separately, and one day posttranscription, 293T cells were treated with MG132 (6 h), followed by CO-IP using the anti-LEF1 antibody. Then, western blot analysis was performed. (g) lncRNA OXCT1-AS1 stably depleted A549 cells were treated with MG132 for 6 h, followed by CO-IP using the anti-LEF1 antibody; then, western blot was performed. (h–j) Lysates from A549 cells with NARF knockdown or NARF overexpression were used to detect the levels of NARF and LEF1 by western blot analysis. (k, l) 293T cells were transfected with Flag-NARF, HA-LEF1, and sh-lncRNA, followed by treatment with MG132 (6 h); next, cell lysates were immunoprecipitated using the antibody against Flag or HA. The precipitates and inputs were analyzed by western blot analysis. (m, n) NARF expression in H1299 and A549 cells with lncRNA OXCT1-AS1 knockdown was detected by western blot.

To elucidate the potential mechanisms underlying the role of lncRNA OXCT1-AS1 in regulating LEF1 stability, we investigated the ubiquitination sites of LEF1 using the Protein Lysine Modifications Database (http://plmd.biocuckoo.org/index.php). The results of Figure 5(a) show that LEF1 had a ubiquitination site at the 54 position, which was close to the binding site of lncRNA OXCT1-AS1 on LEF1 at the 55 position. A549 cells were treated with MG132 to suppress LEF1 degradation. As shown in Figures 5(b) and 5(c), LEF1 levels were significantly reduced following lncRNA OXCT1-AS1 knockdown in the presence of MG132. This suggests that an enhancement of LEF1 stability by lncRNA OXCT1-AS1 is related to proteasomal degradation. Subsequently, we explored the role of lncRNA OXCT1-AS1 on LEF1 expression in the presence of CHX, as shown in Figures 5(d) and 5(e). We found that lncRNA OXCT1-AS1 knockdown shortened the half-life of LEF1. Additionally, lncRNA OXCT1-AS1 overexpression in 293T cells decreased the levels of LEF1 ubiquitination (Figure 5(f)), which was also confirmed in A549 cells (Figure 5(g)). To further investigate the mechanism by which lncRNA OXCT1-AS1 regulates LEF1 ubiquitination, E3 ubiquitin-ligase NARF was knocked down and overexpressed in A549 cells. As shown in Figures 5(h)–5(j), NARF knockdown resulted in a significant increase in LEF1 levels (*P* < 0.01); however, NARF overexpression resulted in a remarkable decrease (*P* < 0.01). Additionally, lncRNA OXCT1-AS1 overexpression weakened the interaction between LEF1 and NARF in A549 cells, including NARF coimmunoprecipitating less LEF1 and LEF1 coimmunoprecipitating less NARF (Figures 5(k) and 5(l)). Interestingly, downregulation of lncRNA OXCT1-AS1 did not affect the levels of NARF (*P* > 0.05), as shown in Figures 5(m) and 5(n). Therefore, these results confirm that lncRNA OXCT1-AS1 abrogates NARF-mediated LEF1 ubiquitination by blocking the interaction between LEF1 and NARF, preventing LEF1 proteasomal degradation.

### 3.6. LEF1 Is Involved in Migration and Invasion of NSCLC Cells

**Figure 6:**
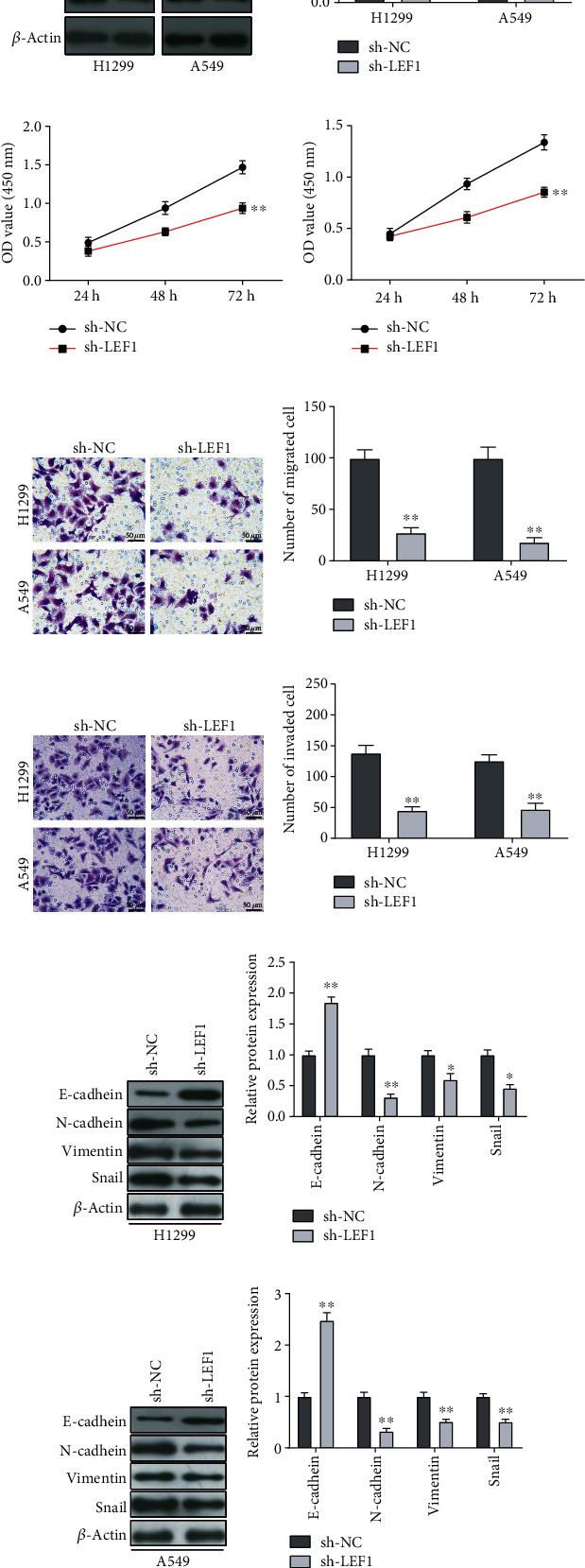
LEF1 knockdown inhibits the migration and invasion of NSCLC cells. (a, b) LEF1 expression in LEF1 stably depleted (sh-LEF1) and negative control (sh-NC) H1299 and A549 cells was assessed by western blot. (c, d) Cell proliferation assays for H1299 (c) and A549 cells (d) transfected with shRNA (sh-LEF1) or negative control (sh-NC) were measured using CCK-8 assays. (e, f) Transwell assays were performed to determine the migration of H1299 and A549 cells. Quantitative data were from 5 fields; scale bar = 50 *μ*m. (g, h) Transwell assays were used to assess the invasion of H1299 and A549 cells. Quantitative data were from 5 fields; scale bar = 50 *μ*m. (i–l) Western blot was performed to measure the level of E-cadherin, N-cadherin, vimentin, and Snail in H1299 and A549 cells, as normalized to the *β*-actin level.

To evaluate whether LEF1 is functionally involved in NSCLC cell metastasis, A549 and H1299 cells with LEF1 knockdown were established. The transfection efficiency of sh-LEF1 was determined by western blot assay (Figures 6(a) and 6(b)). CCK-8 assays showed that the cell viability of H1299 cells with sh-LEF1 transfection was significantly decreased compared with that in the sh-NC group cells (*P* < 0.01, Figure 6(c)). Similar results were obtained in A549 cells (*P* < 0.01, Figure 6(d)). In functional experiments, LEF1 knockdown demonstrated a stronger ability to decrease the migration of H1299 and A549 cells compared with the control cells (Figures 6(e) and 6(f)). Importantly, sh-LEF1 markedly inhibited the invasion of H1299 and A549 cells (Figures 6(g) and 6(h)). Further, we investigated the correlation between LEF1 and proteins associated with epithelial-mesenchymal transition. Western blot assay revealed that LEF1 knockdown significantly downregulated the expression of N-cadherin, vimentin, and Snail, while significantly upregulating E-cadherin expression, in H1299 and A549 cells (Figures 6(i)–6(l)).

### 3.7. LEF1 Is Involved in Metastasis of NSCLC

**Figure 7:**
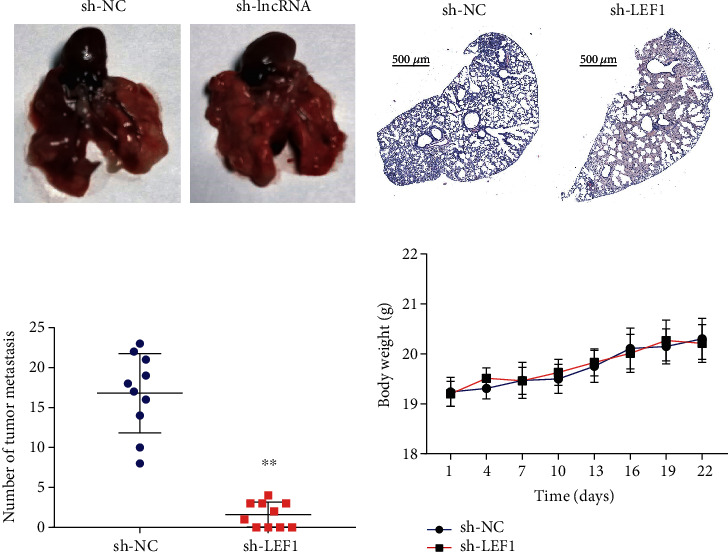
LEF1 knockdown inhibits NSCLC metastasis. (a) The images of metastatic nodules in the lungs of nude mice after tail vein injection with LEF1-downregulated and negative control A549 cells. (b) H&E staining in nude mice after tail vein injection with LEF1-downregulated and negative control A549 cells. (c) The average number of metastatic nodules in the lungs in the tail vein injection model. (d) The body weights of nude mice were measured.

To investigate the possibility of acquired tumor metastasis *in vivo*, LEF1-downregulated and negative control A549 cells were injected into the tail vein of mice. LEF1 knockdown significantly inhibited lung metastasis (Figures 7(a)–7(c)). H&E staining indicated that the number of metastatic nodules in the lung was obviously lower in the sh-LEF1 group than in the sh-NC group (Figures 7(b) and 7(c)). Interestingly, the body weights of mice were shown to be stable (Figure 7(d)). These results collectively suggest that LEF1 plays a role in NSCLC cell metastasis.

## 4. Discussion

The 5-year survival rate of NSCLC is lower than 15%, primarily because of the occurrence of distant metastatic disease [23, 24]. Protein modification is closely associated with metastasis in NSCLC [25]. Among the various complex regulatory networks of cancer metastasis, lncRNAs also play a key role in regulating tumor fate [26]. Despite the rapidly increasing number of lncRNAs with growing knowledge about their underlying mechanisms, studies of lncRNAs in NSCLC metastasis remain inadequate. Here, we characterize, for the first time, the functions of lncRNA OXCT1-AS1 in NSCLC metastasis and the mechanisms involved. Our findings identify potential novel therapeutic targets for cancer metastasis.

**Figure 8:**
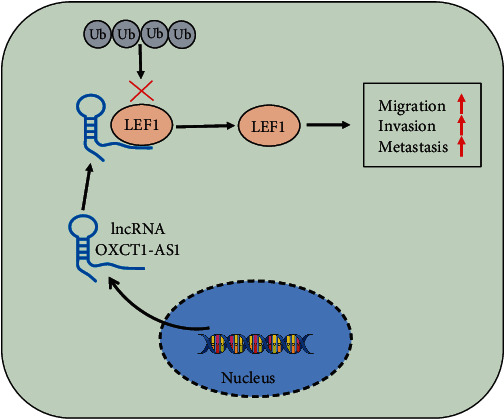
A schematic illustration of the role and molecular mechanism underlying lncRNA OXCT1-AS1 in promotion of NSCLC metastasis. lncRNA OXCT1-AS1 stabilized LEF1 by blocking NARF-mediated ubiquitination and subsequently promoted migration and invasion of NSCLC.

In this study, we demonstrated that knockdown of lncRNA OXCT1-AS1 inhibited the invasion and migration in NSCLC cells. Additionally, the function and pathological significance of lncRNA OTUD6B-AS1 in NSCLC metastasis were also confirmed *in vivo*. Subsequently, we investigated the potential molecular mechanism by which lncRNA OXCT1-AS1 maintains LEF1 stability, and found that this involves the blockade of NARF-mediated LEF1 ubiquitination and proteasomal degradation *in vitro*. Moreover, we found that LEF1 stimulated cell migration and invasion *in vitro* and *in vivo* (Figure 8).

An increasing number of studies have demonstrated that lncRNA is involved in tumorigenesis and development of NSCLC. For example, results from Jiang et al.'s study [27] revealed that the lncRNA HOTAIR contributes to tumorigenesis and metastasis in NSCLC by upregulating miR-613 expression. Therefore, the identification of lncRNA signatures may be of clinical value in the diagnosis, treatment, and prognostic prediction of NSCLC. Recently, a study by Chen et al. [12] highlighted the important roles of lncRNA OXCT1-AS1 in the complex molecular processes that contribute to bladder cancer cell aggressiveness. lncRNA OXCT1-AS1 imprinted 1.7 kb is highly expressed in bladder cancer, metastastic lymph node tissues, and multiple bladder cancer cell lines [12]. A key finding of our study was that the expression of lncRNA OXCT1-AS1 was significantly enhanced in NSCLC cells compared with that in normal lung epithelial cells, especially in the H1299 and A549 cell lines, suggesting the clinical significance of this lncRNA in NSCLC. This observation is additionally consistent with Chen et al.'s report [12].

Extensive research has demonstrated the crucial roles of lncRNAs in NSCLC metastasis [28, 29]. lncRNA PCAT6, a well-studied lncRNA, contributes to tumorigenesis and metastasis in NSCLC by binding to EZH2 [30]. Furthermore, results from He et al.'s study [31] showed that lncRNA AFAP1-AS1, an oncogene, promotes cell migration by increasing AFAP1 expression in NSCLC. In this study, loss-of-function experiments revealed that depletion of lncRNA OXCT1-AS1 strongly inhibited NSCLC cell migration and invasion *in vitro*. Furthermore, *in vitro* function assays showed that lncRNA OXCT1-AS1 knockdown significantly reduced the number of metastatic nodules in the lung. These data confirmed that lncRNA OXCT1-AS1 induces NSCLC metastasis. Silencing lncRNA OXCT1-AS1 may represent a promising strategy to inhibit NSCLC metastasis.

lncRNAs have been shown to exert their functions through a variety of mechanisms, by directly interacting with proteins, mRNAs, and/or microRNAs [12, 32]. In the present study, we performed cell nuclear and cytoplasmic RNA isolation and found that lncRNA OXCT1-AS1 was enriched in the cytoplasm, which suggests that lncRNA OXCT1-AS1 plays a role in metastasis in a posttranscriptional-dependent manner. However, the mechanisms by which lncRNA OXCT1-AS1 induces metastasis remain largely unknown, with numerous studies proposing that protein regulation is involved [33, 34]. Using bioinformatics databases, 429 downstream targets were predicted. Considering that transcription factor LEF1 is reported to be involved in lung cancer metastasis [20], and there is a steric hindrance effect that the binding of lncRNA OXCT1-AS1 to the 55 position of LEF1 affects the ubiquitination of LEF1 at the 54 position, we selected LEF1 as a target. Interestingly, we found that lncRNA OXCT1-AS1 knockdown resulted in the attenuation of LEF1 protein expression. LEF1, a downstream factor of the WNT/*β*-catenin pathway, regulates gene transcription independently [35]. Extensive research has shown that LEF1 plays an essential role in the epithelial-mesenchymal transition by activating the transcription of N-cadherin, vimentin, and Snail [34, 35]. Bleckmann et al. [36] reported that LEF1 overexpression correlates with poor prognosis in cerebral metastasis of lung adenocarcinomas. Therefore, we hypothesized that lncRNA OXCT1-AS1 maintains LEF1 stability to contribute to NSCLC metastasis. To verify this hypothesis, validation experiments were conducted, and showed that lncRNA OXCT1-AS1 directly interferes with LEF1 through its fragments 800 nt and 1201 nt, supporting our hypothesis. These findings also suggest that LEF1 stability is maintained by lncRNA OXCT1-AS1 at the posttranslational level.

Accumulating evidence shows that the stability of LEF1 can be controlled by its ubiquitination [37] and sumoylation [38]. Interestingly, recent research indicates that lncRNAs are capable of regulating protein ubiquitination [34, 39]. For example, Tang et al. [40] found that the lncRNA GLCC1 stabilizes c-Myc by blocking ubiquitination via direct interaction with the HSP90 chaperone protein. In this study, we found that lncRNA OXCT1-AS1 blocked LEF1 ubiquitination, thereby preventing LEF1 proteasomal degradation. Previous studies have indicated that nemo-like kinase-associated ring finger protein NARF regulates the ubiquitylation and degradation of LEF1 [41]. Therefore, we explored the role of NARF in lncRNA OXCT1-AS1-mediated LEF1 stability, and found that lncRNA OXCT1-AS1 knockdown facilitates the interaction between NARF and LEF1. These findings show that lncRNA OXCT1-AS1 blocks NARF-mediated LEF1 ubiquitination by inhibiting the interaction between NARF and LEF1, thereby preventing LEF1 proteasomal degradation. Our findings are largely consistent with previous studies, in which constitutively activated STAT5a was shown to recruit the E3 ubiquitin-ligase NARF to LEF1 and lead to LEF1 ubiquitination and degradation [42]. These findings highlight the important role of lncRNA OXCT1-AS1 in NSCLC metastasis. Although lncRNA OXCT1-AS1 stabilized LEF1 in NSCLC, the other regulatory mechanisms for this lncRNA remain undefined.

An increasing number of studies indicate that LEF1 is essential for invasion, migration, and epithelial-mesenchymal transition in cancer cells [43]. For example, Rosmaninho et al. [44] found that Zeb1 potentiates LEF1 transcription to promote glioblastoma cell invasion. In the present study, we observed that LEF1 knockdown markedly reduced the migration and invasion of NSCLC cells. These functional roles of LEF1 were further confirmed by loss-of-function assays *in vivo*. In line with previous findings in esophageal squamous cell carcinoma [21], these results confirm that lncRNA OXCT1-AS1 promotes NSCLC metastasis by stabilizing LEF1.

In conclusion, the present study reveals, for the first time, that upregulation of the lncRNA OXCT1-AS1 induces NSCLC metastasis *in vitro* and *in vivo*. Mechanistically, our findings demonstrate that lncRNA OXCT1-AS1 blocks NARF-mediated LEF1 ubiquitination by inhibiting the interaction between LEF1 and NARF, thereby preventing LEF1 proteasomal degradation. Furthermore, LEF1 was confirmed to be involved in NSCLC metastasis. These findings establish a foundation for the application of lncRNA OXCT1-AS1 in diagnosis, prognostic evaluation, and therapy of NSCLC metastasis. Although these findings may lack clinical evidence, our study still have implications for treatment strategies of NSCLC metastasis.

## Data Availability

All data generated or analyzed during this study are included in this published article. The datasets generated and/or analyzed during the current study are available from the corresponding author on reasonable request.
